# Tritordeum: Creating a New Crop Species—The Successful Use of Plant Genetic Resources

**DOI:** 10.3390/plants10051029

**Published:** 2021-05-20

**Authors:** Carmen M. Ávila, Cristina Rodríguez-Suárez, Sergio G. Atienza

**Affiliations:** 1 Área Genómica y Biotecnología, IFAPA—Centro Alameda del Obispo, Apdo 3092, 14080 Córdoba, Spain; carmenm.avila@juntadeandalucia.es; 2 Instituto de Agricultura Sostenible (CSIC), Alameda del Obispo, s/n, E-14004 Córdoba, Spain; crodriguez@ias.csic.es

**Keywords:** tritordeum, *Hordeum chilense*, pre-breeding, genetic resources

## Abstract

Hexaploid tritordeum is the amphiploid derived from the cross between the wild barley *Hordeum chilense* and durum wheat. This paper reviews the main advances and achievements in the last two decades that led to the successful development of tritordeum as a new crop. In particular, we summarize the progress in breeding for agronomic performance, including the potential of tritordeum as a genetic bridge for wheat breeding; the impact of molecular markers in genetic studies and breeding; and the progress in quality and development of innovative food products. The success of tritordeum as a crop shows the importance of the effective utilization of plant genetic resources for the development of new innovative products for agriculture and industry. Considering that wild plant genetic resources have made possible the development of this new crop, the huge potential of more accessible resources, such as landraces conserved in gene banks, goes beyond being sources of resistance to biotic and abiotic stresses. In addition, the positive result of tritordeum also shows the importance of adequate commercialization strategies and demonstrative experiences aimed to integrate the whole food chain, from producers to end-point sellers, in order to develop new products for consumers.

## 1. Introduction

Rice, maize and common wheat are the most important crops for human consumption in the world. Both rice and maize are diploids, but bread wheat is an allohexaploid (2n = 6x =42, AABBDD) derived from the cross between *Triticum turgidum* (AABB) and *Aegilops tauschii* (DD) [1]. The allohexaploid genome structure of bread wheat is, in part, responsible for the adaptability of this crop to a wide range of climatic conditions [1].

The wide adaptability of polyploids is an interesting feature for breeding, but alloploidy has not been generally exploited by breeders since it is usually associated with sterility. The first triticale was obtained by Rimpau in 1888, after spontaneous chromosome doubling of hybrids from crosses between bread wheat and rye Rimpau, 1891 (as cited in [2]). The development of triticale from the first cultivars released in the 60 s to our days, exemplifies the possibilities of alloploidy for the development of new crops (reviewed by [2]).

The success of triticale renewed the interest of developing new synthetic amphiploids between barley and wheat. Plant breeders had been interested in crossing both crops since the beginning of the 20th century (reviewed by [3]), but fertile amphiploids were only obtained when the wild barley *Hordeum chilense* Roem. et Schultz. was used. This new species was named tritordeum (× *Tritordeum martini* A. Pujadas) [4]. Octoploid [5] and hexaploid [6] tritordeums were obtained from the crosses between *H. chilense* (as mother) and common or durum wheat as pollen donors, respectively. Both tritordeums were initially considered for breeding but the hexaploid became the species of choice since octoploid tritordeums showed a high chromosome instability. A similar situation happens in triticale. Although different ploidy levels have been developed and studied, only hexaploid triticale (× *Triticosecale* Wittmack, 2n = 6x = 42) has commercial application (reviewed by [2]).

After two decades of breeding, the potential of tritordeum was clear [3,7]. Hexaploid tritordeum was perceived as an interesting new crop with a similar role to bread wheat in the food industry and with potential as a bridge to transfer useful traits from *H. chilense* to wheat. However, tritordeum breeding still faced significant problems to become a new crop. The most important limitations were the persistence of traits from the wild progenitor, the lack of molecular tools for the effective study and utilization of traits of interests inherited from *H. chilense* and the competition in the food industry with bread wheat-derived products. In this review, we summarize the findings and achievements of the last 20 years which have allowed the successful development of hexaploid tritordeum as a new crop.

## 2. Progress in Breeding for Agronomic Performance

Tritordeum showed a promising potential at the beginning of this century but it still faced significant problems including the retention of traits from its wild progenitor. In first place, tritordeums had brittle rachis. This is an important adaptive trait in the wild that allows an efficient seed dispersion but it is a non-deal trait for agriculture. In addition, tritordeum breeding lines also presented tenacious glumes that interfere with threshing. The combination of both traits resulted in high yield losses during harvesting and constituted a barrier for tritordeum cultivation and commercialization. The improvement of both traits was addressed in the breeding program through the research project ‘Breeding of tritordeum’ (AGL2005-01381) using two different approaches. The first consisted in the utilization of mutagenic substances in seeds of both *H. chilense* and tritordeum looking for tough rachis mutant phenotypes. This approach was unsuccessful (unpublished results) but allowed the identification of imidazolinone resistant tritordeums due to the mutation in the acetohydroxiacid synthase locus (a single Ser-Asn627 substitution) [8]. This mutation would facilitate an efficient weed management as happens with Clearfield^®^ wheat varieties [9]. Furthermore, this mutated locus has been successfully transferred to durum wheat and constitutes and additional source of resistance to imidazolinone herbicides available in this species [10].

The second approach consisted in a crossing program between hexaploid tritordeum and common wheat. The aim of this research was to obtain free threshing lines throughout the development of chromosome substitution lines. Free threshing is determined by the *Q* locus in chromosome 5A [11,12] and it controls pleiotropically other traits including glume tenacity and rachis fragility. The extensive search in the breeding program allowed the identification of three hexaploid tritordeum lines (HT374, HT376 and HT382) with free threshing ability [13]. Molecular and cytogenetic characterization of these lines showed that both HT374 and HT376 carried a substitution 5D/(5H^ch^), which suggested the role of an homoeologous *Q* factor located in 5H^ch^. The molecular characterization of the *Q* gene in wheat [12] allowed the study of this transcription factor in tritordeum. The cloning and characterization of the *Q* gene from *H. chilense* showed that this gene was absent in HT374 while it was present in HT382 [14]. These results suggest that the free-threshing ability of HT374 was derived from the lack of the AP2-like gene from *H. chilense* in 5H^ch^ [14]. On the other hand, the characterization of the breeding line HT382 revealed a double substitution 1D/(1H^ch^), 2D/(2H^ch^). The *Tenacious glume* (*Tg*) locus is located in chromosome 2D [15] and, thus, the substitution of chromosome 2H^ch^, eliminating the homoeologue *Tg* locus from *H. chilense*, was considered as the cause of the free threshing ability of HT382 [13].

Regarding yield and agronomic performance, tritordeum breeding lines showed similar behaviour to wheat and triticale elite cultivars under low water conditions [16]. However, further efforts to achieve extended grain filling period and earlier anthesis were required [16]. The continuous breeding pressure allowed the selection of lines with good threshing ability, without any bread wheat chromosomes and with yield levels similar to wheat in regions with temperate winters (South of Spain). As a result, two tritordeum varieties, ‘Aucan’ (grant number 35093), and ‘Bulel’ (grant number 40872), were registered in the Community Plant Variety Office.

Tritordeum, including ‘Bulel’, seems better adapted to organic farming than durum wheat since it shows an increase in the below ground community of the Bacteroidetes phylum and better grain quality than durum wheat [17]. However, it has a lower grain yield [17]. However, it is important to note that new advanced lines of tritordeum outperform ‘Aucan’ and ‘Bulel’ for yield performance [18]. Table 1 summarizes the agronomic performance data available for tritordeum over time.

Tritordeum can also be used as a bridge species to transfer useful traits from *H. chilense* to wheat. *H. chilense* shows resistance to many diseases which could be exploited for wheat breeding [20]. For instance, tritordeum is resistant to Septoria leaf blotch (STB) due to the gene(s) located on chromosome 4H^ch^ of *H. chilense* (reviewed by [20]). The evaluation of resistance to STB on naturally infected trials allows the identification of genes effective against the local isolates, but they may not be effective if diversity at avirulence loci exists [21]. Field trials evaluation in the Czech Republic confirmed the high average resistance of tritordeums for Septoria leaf blotch [22]. The confirmation of the resistance of tritordeum to septoria leaf blotch in a completely different environment from Córdoba (Spain), where the initial resistance tests were conducted, is a good sign showing the potential of the resistance against this pathogen. Substitution lines for *H. chilense* chromosome 4H^ch^ into durum wheat have been obtained [23]. Although their performance against STB was not evaluated, they were considered a valuable tool for durum wheat breeding [23]. Similarly, many examples of successful introgression of *H. chilense* into wheat genetic background are available [24,25,26,27,28,29,30,31,32,33,34]. *H. chilense*-wheat translocation lines have been developed (Table 2). Nevertheless, to our knowledge, the transference of these introgressions into elite wheat material is still pending.

In addition, tritordeum is also considered a potential source to introgress genes for the combined stress of drought and salinity, as well as to each of these stresses separately [35] and for the development of hybrid wheat using a cytoplasm male sterility (msH1 system) [26,27,36]. The utilization of msH1 system for the production of hybrid bread [37] and durum wheat [38] is a clear example of the benefits of tritordeum for the improvement of wheat.

**Table 2:** Translocation lines (TL) of *H. chilense* in bread wheat (BW) and durum wheat (DW).

Genetic Stock	Chromosome	Main Traits
TL in BW [25]	2H^ch^	Carotenoid [39] and PPO1 and PPO2 genes [40];
TL in BW [30]	3H^ch^	eLcy [39]; carotenoid content [41]
TL in DW [23]	4H^ch^	STB and greenbug resistance [20]; salinity [42]
TL in BW [43]	5H^ch^	Mildew and greenbug resistance [20]; Hordeoindolines [43]; Salt tolerance [42]; Carotenoid genes [39]
TL in BW [27]; TL in DW [38]	6H^ch^	Fertility restoration [27,34,37,38]
TL in BW [28,29,31]	7H^ch^	Carotenoid content [31,44,45]; carotenoid esterification [45,46]; mildew and greenbug resistance [20]; waxy protein [47]

## 3. Impact of Molecular Markers in Genetics and Breeding

The properties of tritordeum are influenced to a great extent by *H. chilense* genome. In this context, the development of genetic studies and the characterization of plant breeding materials, including introgression lines, could benefit from the application of molecular markers. Progress in genomics during the last two decades have made possible the genotyping with thousands of markers with a low cost per data and in a short time. However, the situation was very different two decades ago. No DNA markers were available for *H. chilense* at the early stages of tritordeum breeding [3]. Thus, a considerable effort was employed for the development of molecular markers suitable for genetic studies in *H. chilense* and for the development of marker assisted introgression of *H. chilense* chromatin into wheat background using RAPDs, AFLP, SSR and RFLP (reviewed by [48]. RAPDs and AFLP markers allowed the first mapping studies in *H. chilense* [49,50,51]. However, the lack of enough markers for comparative studies among species, such as SSR of RFLP, constituted a serious drawback in order to exploit the knowledge generated in related cereals. Genomic studies in barley allowed the development of EST markers in a much larger scale than previously known. The transferability of these markers to *H. chilense* [51,52,53] constituted a qualitative jump for the identification of *H. chilense* chromosomes in wheat background. Indeed, these markers have been successfully used for the identification of *H. chilense* chromosomes in tritordeum [13] and for the identification of *H. chilense* chromosomes during the development of wheat-*H. chilense* genetic stocks for chromosomes 1H^ch^ [24]; 2H^ch^ [25]; 3H^ch^ [30]; 4H^ch^ [23,32], 6H^ch^ [27] and 7H^ch^ [28,29].

The synthesis of new allopolyploids results in the elimination of chromosome- and genome-specific sequences contributing to the diploid-like meiotic behaviour [54]. Furthermore, this elimination is non-random and directional, and contributes to the diploid-like behaviour of the amphiploids [54]. The existence of these rearrangements was studied in tritordeum with different types of markers including inter-retrotransposon amplified polymorphism (IrAP), retrotransposon-microsatellite amplified polymorphism (REMAP) and Start Codon Targeted (SCoT) polymorphisms [55,56]. The elimination of *H. chilense* sequences, as deduced from the fact that the majority of SCoT markers were derived from wheat instead of from *H. chilense*, reinforced the potential of tritordeum as a new crop [55].

Despite the progress in the application of molecular markers, advances in sequencing techniques and microarray-based markers constituted another qualitative change for genetic studies in *H. chilense* and tritordeum. In particular, DArT markers allowed the development of thousands of markers widely distributed throughout *H. chilense* genome. These markers were used for the construction of a genetic map with a good coverage [57] and for the genetic characterization of tritordeum breeding lines [58]. Furthermore, this new genetic map was completed with barley ESTs [59] and COS markers [60], providing the bases for preliminary macro-synteny studies of *H. chilense* with other Triticeae species. The genomic coverage obtained with this genetic map made possible the location of candidate genes including carotenoid and polyphenol oxidase genes [39,40] and the mapping of the fertility restoration locus in chromosome 6H^ch^ [33] in the wheat-msH1 cytoplasmic male sterility system useful for hybrid wheat production [27]. The correspondence of the location of all these genes in *H. chilense* with their homoeologues in other Triticeae species, suggested a good degree of collinearity between *H. chilense* and the rest of the tribe members. After this, the development of DArTSeq markers in *H. chilense*, along with the availability of the barley genome sequence in public repositories [61], made it possible to study the synteny relations between *H. chilense* and barley in detail [62]. In general, *H. chilense* shows a good degree of collinearity with barley with the exception of a major rearrangement in chromosome 7H^ch^, where *H. chilense* carries a reciprocal translocation between the distal part of this chromosome [62]. The break of synteny at 7H^ch^ was suspected since the main locus for endosperm carotenoid content in *H. chilense* has been located in chromosome 7H^ch^S [44], while the orthologous was located in chromosome 7BL in durum wheat [63]. DArTSeq markers have been used for Genome-Wide Association Scan studies in *H. chilense* [62] and they have also contributed to the study of the cytoplasmic male sterility msH1 system [27] for the production of hybrid wheat. In particular, DArTSeq markers allowed the characterization of an acrocentric chromosome carrying the restorer-of-fertility gene [33] as a previous step for the identification of the candidate gene [37].

## 4. Progress in Quality and Potential for the Development of New Innovative Food Products

Although durum wheat is the male parent of hexaploid tritordeum, the grain texture of tritordeum is similar to that of bread wheat [64]. This quality parameter is controlled in wheat by the puroindoline genes (*Pina-D1* and *Pinb-D1*) located on chromosome 5D [65] and the homoeologue hordoindoline genes *Hina* and *Hinb* in barley. *Hina-Hch1* and *Hinb-Hch1* genes in *H. chilense* are very similar to *Pin* genes of bread wheat [66], which may explain the soft grain texture of tritordeum. Indeed, the addition of chromosome 5H^ch^ to bread wheat resulted in the enhancement of grain softness [67]. This makes tritordeum flour more adequate for the production of products similar to those obtained from bread wheat. Accordingly, High Molecular Weight (HMW) glutenin subunits were considered a primary target for tritordeum breeding due to their high influence on breadmaking quality [68]. Two alternative approaches were applied: transgenic and conventional breeding. The transgenic alternative allowed the development of tritordeum lines expressing HMW genes 1Ax1 and 1Dx5 [69,70]. On the other hand, chromosome substitution or translocation lines with the HMW glutenin subunits Dx5 + Dy10 were obtained by conventional methods [71]. The chromosome substitution lines obtained showed a similar agronomic performance than the euploid tritordeum, but they had a much higher gluten strength due to the addition of HMW glutenin subunits 1D [72].

The breadmaking quality of tritordeum is also influenced to a great extent by the *H. chilense* genome (reviewed by [3]). This promoted the study of the variability for endosperm storage proteins in the *H. chilense* accessions used to develop primary tritordeums [73,74,75], along with the diversity in the natural populations of the species [76]. Further studies focused on the effect of these proteins in breadmaking quality in tritordeums [77,78]. In summary, these studies revealed a wide diversity for storage proteins potentially useful for both tritordeum and wheat breeding, which could provide new functionalities not found in other cereals. Indeed, tritordeum has significantly lower levels of ω-gliadins in flour and levels of gluten around 50% lower than wheat [79]. Accordingly, tritordeum is considered an interesting choice to people wishing to reduce their gluten intake, although it is not suitable for patients suffering coeliac disease [79]. Furthermore, tritordeum bread has been recommended for a subset of non-celiac wheat sensitivity patients who do not need strict exclusion of gluten from their diet [80].

The increasing demand of healthier foods, including whole grain-derived foods, has promoted the investigation of other health related traits in tritordeum. Phenolic compounds are the main group of phytochemicals in barley grain and their main interest is due to their strong antioxidant power and their association to certain diseases prevention [81]. Considering that tritordeum expresses the properties of both barley and wheat, its phenolic content and profile was investigated [82]. A great variability for phenolic compounds content was reported, ferulic acid being the main one that happens in wheat [82]. However, comparative studies with wheat and barley showed that advanced lines of tritordeum have a similar total phenolic content to wheat but much lower than barley [83]. Phenolic content has not been a target in tritordeum breeding program and, thus, it might be possible that valuable diversity for this trait remains hidden in pre-breeding materials or in *H. chilense* accessions. However, total phenolic content in tritordeum was around half the reported in barley [83] and, thus, it is not likely that tritordeum could outperform barley as a natural source for these compounds.

Accumulation of compounds such as tocols [84] or polysaccharides (arabinoxylans and β-glucans) [85] has been also studied in tritordeum, along with the potential for accumulation of micronutrients as Selenium in grain [86]. Furthermore, the essential role of Selenium in animal and human nutrition promoted the evaluation of selenium fertilized tritordeum in relation to conventional dietary supplements of this micronutrient in laying hens [87]. The improvement of egg quality due to Se-enriched tritordeum suggests that selenium-fertilized tritordeum may be an interesting alternative for animal feeding [87].

Tritordeum can be also used for cake [88] and beer production [89]. Indeed, tritordeum and barley malts yielded comparable values for the majority of technological parameters including alcohol content, although tritordeum malts produced a slight acidification effect, a lower level of glucose and a higher amount of free amino nitrogen [89]. Besides, tritordeum malt did not cause any technological problem during the different stages of beer production and, thus, it is considered that it has a high potential for the brewing industry [89]. Furthermore, the utilization of brewers’ spent grain from tritordeum, the major by-product of the brewing industry, may increase the nutritional potential of durum wheat pasta [90], by improving total antioxidant capacity, total dietary fibre and β-glucans and without compromising the sensory aspects of pasta [90]. All these findings show the potential of tritordeum for the development of food products with new functionalities.

Regarding health-related compounds, carotenoid content has been the most extensively studied due to its importance on the appearance of tritordeum products. The intensive yellow colour of tritordeum flour constitutes an important differential characteristic compared to bread wheat derived products [3]. This trait could be perceived as detrimental since white flour is usually preferred for breadmaking from bread wheat. As a consequence, initial studies confirmed the lack of effect of yellow colour in relation with the baking performance [3]. The high carotenoid content in the endosperm is responsible for the golden coloration of tritordeum products and it confers a clear differentiation from standard bread wheat products. Instead of a detrimental trait, the high carotenoid content was considered a potential commercial advantage. This motivated the study of the genetic bases of carotenoid content, which resulted in the identification of a QTL in chromosome 2H^ch^ [50], the selection of new genotypes with high carotenoid content such as HT621 [91] and the development of selection tools useful for the breeding program [92]. The genes responsible for carotenoid content in tritordeum and wheat were unknown at the time although they have been located in chromosomes 7H^ch^ [44] and 7B [63]. Thus, a candidate gene approach using rice as a model species and the gene *Phytoene synthase 1* was performed. Our results proved that *Psy1* was located in chromosome 7H^ch^S in *H. chilense* and 7A and 7B in durum wheat [93]. Furthermore, the diagnostic marker developed for *Psy1_Hch* [93] was successfully used for marker assisted selection of *Psy1_Hch* in bread wheat-*H. chilense* genetic stocks [28,45]. The cloning and heterologous expression in bacteria of *Psy1_Hch* confirmed the functionality of this gene [94] and its potential for the enhancement of carotenoids in wheat.

Further transcriptomic experiments showed that both *Psy1* and *e-Lcy* (Lycopene epsilon cyclase) were upregulated between 18 and 25 days after anthesis in tritordeum, while their homoeologue genes in durum wheat were downregulated [41]. The differences in the expression profile between tritordeum and durum wheat were associated with the differences in carotenoid content between both species [41]. The development of translocation lines of *H. chilense* 7H^ch^S into bread wheat resulted, as expected, in the increase in endosperm carotenoid content due to the presence of *Psy1_Hch* [31].

In addition, tritordeums have a high proportion of carotenoid esters in contrast with durum wheat [95]. Esterification is a common way to accumulate carotenoids in plants [96]. Thus, it was hypothesized that the activation of the carotenoid pathway in tritordeum during grain development may be associated with the synthesis of carotenoid esters and the production of a metabolic sink. However, this hypothesis was not confirmed since no lutein esters are produced before 36 days after anthesis [97]. Nevertheless, carotenoid esterification can contribute to the accumulation of lutein in tritordeum endosperm by limiting carotenoid degradation in later stages [97].

The importance of esterified carotenoids goes beyond their role in carotenoid accumulation since they have a higher stability than free carotenoids [98,99]. Higher carotenoid retention has been observed during post-harvest storage due to esterification [98,100,101,102] and, thus, the increase in carotenoid esterification is a good target for the improvement of carotenoid retention in the food chain. This is relevant for tritordeum since this species has a high proportion of carotenoids in the esterified form in the endosperm [95,97,103], with a 3-fold higher content in the endosperm compared to the germ [104].

The potential of esterification for the improvement of carotenoid retention through the food chain has increased the interest on this trait despite these results are not confirmed at high-temperature regimes [105]. The identification of the xanthophyll acyl transferase (*XAT-7D*), responsible for carotenoid esterification in common wheat, opens new possibilities for marker assisted selection [106]. In fact, this gene is being transferred from common to durum wheat at present [107]. In tritordeum, carotenoid esterification is due to the *H. chilense* genome [108]. In particular, candidate genes at chromosome 7H^ch^ were identified by physical mapping and DArTSeq markers [109]. Recently, a GDSL esterase/lipase (XAT-7Hch), orthologue of XAT-7D, has been identified as the main responsible for lutein esterification in *H. chilense*/tritordeum [46]. As happens with XAT-7D, this gene can be used for wheat breeding through a marker assisted selection strategy with a diagnostic marker already available [46].

Table 3 summarizes the main quality attributes of tritordeum grain.

The high carotenoid content of tritordeum has been used to widen the interest in this ‘Golden Cereal’ (https://www.tritordeum.com/?lang=en#whatis, accessed on 20 May 2021). Agrasys S.L., Barcelona, Spain, a spin-off of the Spanish High Council for Scientific Investigations (CSIC), has benefited from the commercial exploitation of tritordeum, as it has held the exclusive commercial rights of tritordeum since 2006. The registration of the tritordeum varieties ‘Aucan’ and ‘Bulel’ at the Community Plant Variety Office, along with the commercialization effort developed by Agrasys, has made the expansion of tritordeum possible to many countries (https://www.tritordeum.com/ww/?lang=en, accessed on 20 May 2021); more importantly, it has made tritordeum products available to consumers. At present, there is a complete food chain comprised of farmers, millers, bakers and sellers for the development of tritordeum products.

## 5. Final Remarks

The success of tritordeum as a crop offers important lessons. In the first place, it shows the importance of the effective utilization of plant genetic resources, including wild species, for the development of innovative products for agriculture and food industry. The utilization of plant genetic resources must be encouraged and promoted, since they hold the key for further success in the current scenario of climate change, and to provide the food industry with new products to meet the consumer’s preferences. Considering that the use of wild germplasm, without any adaptation to agriculture, has been used to develop a new crop, plant genetic resources conserved in germplasm banks have huge potential for plant breeding that goes beyond being sources of resistance to pest and diseases. The development of introgressions of *H. chilense* into common and durum wheat genetic backgrounds holds potential for the improvement of wheat. However, the transference of these introgressions into elite wheat material is still pending.

In second place, the development of innovative products is not enough to produce a significant impact in the food industry. In this context, the efforts carried out by Agrasys S.L. have promoted the interest of producers, millers and consumers in tritordeum, through adequate commercialization strategies and demonstrative experiences, which have allowed the consumption of tritordeum in an ever-growing list of countries around the world.

## Figures and Tables

**Table 1:** Agronomic performance of tritordeum lines over time.

		TKW		Yield		
Plant Material ^1^	Location ^2^	Value (g)	Relative Performance ^3^	Value (kg/Ha)	Relative Performance	Reference
First HTs		36.0	66.2	no data		[6]
Primary tritordeums	Córdoba (Spain)	35.4	65.2	n.d.		[2]
Breeding lines	Gimenells (Spain)	33.4	64.5	2262	52.5	[16]
Breeding lines	Tal-Amara (Lebanon)	27.2	64.7	2744.5	54.6	[16]
Breeding lines	Córdoba (Spain)	31.5	77.6	3173.3	73.5	[16]
Breeding lines	Granada (Spain)	33.8	85.1	2364.5	58.9	[16]
Breeding lines	Nabeul (Tunisia)	22.9	66.8	1257.5	54.7	[16]
Advanced lines	Larisa (Greece) 1st	29.6	103.5	4327.5	59.0	[18]
JB1	Larisa (Greece) 1st	31.4	110.0	3666	50.0	[18]
JB3	Larisa (Greece) 1st	28.5	99.8	3211	43.8	[18]
Advanced lines	Larisa (Greece) 2nd	29.8	101.1	3480	85.8	[18]
JB1	Larisa (Greece) 2nd	31.3	106.3	3189	78.6	[18]
JB3	Larisa (Greece) 2nd	28.7	97.5	3196	78.8	[18]
Aucan	Écija	n.d.	n.d.	3404.1	117.3	[19]
Aucan	Jerez (Spain)	n.d.	n.d.	4717.2	107.5	[19]

^1^ JB1 and JB3 were pre-selections of ‘Aucan’ and ‘Bulel’ registered varieties. ^2^ Two seasons (denoted as 1st and 2nd) were considered by [18]. ^3^ Relative performance compared to bread wheat control with the exception of First HTs that are compared to durum wheat parents.

**Table 3:** Grain quality parameters in tritordeum.

Trait	Value	Relative Performance Over Control ^1^	Reference
Carotenoid content (µg/g) (Primary tritordeums)	5.8	4.8-fold increase (DW)	[95]
Carotenoid content (µg/g) (Breeding lines)	9.14	2.8-fold increase (DW)	[41]
Carotenoid content (Bread) (µg/100 g)	357.6	6.5-fold increase (BW)	[19]
Carotenoid esterification (%)	33.8	not detected (DW)	[41]
Gluten content	n.a.	51% reduction (BW)	[79,80]
γ-gliadin epitopes	n.a.	59% reduction (BW)	[79,80]
α-gliadin epitopes	n.a.	77% reduction (BW)	[79,80]
Total tocols (µg/g)	30.2	Similar to BW	[84]
Beta-glucans (% dry matter)	0.6	Similar to BW; 90% reduction (B)	[85]

^1^ DW = durum wheat; BW = Bread wheat; B = Barley.

## Data Availability

No data are contained within the article.
